# Can community action improve equity for maternal health and how does it do so? Research findings from Gujarat, India

**DOI:** 10.1186/s12939-018-0838-5

**Published:** 2018-08-20

**Authors:** Asha S. George, Diwakar Mohan, Jaya Gupta, Amnesty E. LeFevre, Subhasri Balakrishnan, Rajani Ved, Renu Khanna

**Affiliations:** 1 School of Public Health, University of the Western Cape, Private Bag x17, Bellville, Cape Town, 7535 South Africa; 2 School of Public Health, Johns Hopkins University, 615 North Wolfe Street, Baltimore, MD 21205 USA; 3 Faculty of Health Sciences, University of Cape Town, Barnard Fuller Building, Anzio Road, Observatory, Cape Town, 7935 South Africa; 4 Common Health, Rural Women’s Social Education Centre RUWSEC, 61, Karumarapakkam Village, Veerapuram Post, Thirukazhukundram, Kancheepuram, Tamil Nadu 603109 India; 5 Independent Consultant, New Delhi, India; 6 SAHAJ, 13 Krishana Society, Haribhakt Lane, Old Padra Road, Vadodara, Gujarat 390015 India

**Keywords:** Care-seeking, Maternity care, Equity, Accountability, Community monitoring, Report cards, Public-private mix

## Abstract

**Background:**

Efforts to work with civil society to strengthen community participation and action for health are particularly important in Gujarat, India, given that the state has resources and capacity, but faces challenges in ensuring that services reach those most in need. To contribute to the knowledge base on accountability and maternal health, this study examines the equity effects of community action for maternal health led by Non-Government Organizations (NGOs) on facility deliveries. It then examines the underlying implementation processes with implications for strengthening accountability of maternity care across three districts of Gujarat, India. Community action for maternal health entailed NGOs a) working with community collectives to raise awareness about maternal health entitlements, b) supporting community monitoring of outreach government services, and c) facilitating dialogue with government providers and authorities with report cards based on community monitoring of maternal health.

**Methods:**

The study combined qualitative data (project documents and 56 stakeholder interviews thematically analyzed) with quantitative data (2395 women's self-reported receipt of information on entitlements and use of services over 3 years of implementation monitored prospectively through household visits). Multivariable logistic regression examined delivery care seeking and equity.

**Results:**

In the marginalised districts, women reported substantial increases in receipt of information of entitlements and utilization of antenatal and delivery care. In the marginalized and wealthier districts, a switch from private facilities to public ones was observed for the most vulnerable. Supportive implementation factors included a) alignment among NGO organizational missions, b) participatory development of project tools, c) repeated capacity building and d) government interest in improving utilization and recognition of NGO contributions. Initial challenges included a) confidence and turnover of volunteers, b) complexity of the monitoring tool and c) scepticism from both communities and providers.

**Conclusion:**

With capacity and trust building, NGOs supporting community based collectives to monitor health services and engage with health providers and local authorities, over time overcame implementation challenges to strengthen public sector services. These accountability efforts resulted in improvements in utilisation of public sector services and a shift away from private care seeking, particularly for the marginalised.

## Background

Community mobilization is essential in raising awareness of health and health rights, as well as in changing the social norms and inequalities that marginalize women from affirming their health and rights [1]. Community participation is also a key factor underpinning intersectoral action for health, central to addressing the social determinants of health. Despite their instrumental to health and development goals, communities are also social forums vital to governing health systems. Community health committees and score cards play key roles in representing community perspectives, ensuring that community voices are articulated, heard and responded to, so that trust, mutual understanding and respect in health care services can be restored [2, 3]. As a result, when coupled with support from outside resources and actors across health system levels, they can also be instrumental in realizing improvements in service delivery [4].

The World Health Organisation's endorsement of community participation noted that the evidence underpinning its effects on maternity care seeking and health equity is weak [5]. A systematic review found 22 community based efforts to promote awareness of women's rights to maternal health often within a community accountability framework [6]. However, out of these 22 projects, only four measured effects on care seeking [7–10], and across these four studies the underlying implementation processes underpinning accountability interventions were not well described [5, 6, 11].

Several of the stronger case studies in the systematic review are based in India, which has a rich history of non-governmental organization (NGO) and community engagement in health [10, 12–14]. NGOs are a key constituency advising the National Health Mission's community strategies [15–17]. Feminist NGOs have a long history in advocating for health policies and programs to be more responsive to women's health needs and rights [12, 18, 19]. NGOs also serve a strong role in providing alternative models for service delivery based on Alma Ata principles [20]. The People's Health Movement has a strong membership in India and works closely with NGOs and community based organizations in advocating for the right to health. Community based organisations and mass organisations have supported social movements affirming women's rights to livelihoods and health across the country.

With the aim of contributing to the knowledge base on community participation in health in low and middle income countries broadly [21] and more specifically on how community action can improve care seeking and service delivery of maternity services for marginalized communities, this study examines an NGO led project in Gujarat, India, identified in an earlier systematic review [6] as warranting further evaluation. The study assesses the effects of community action on access to facility deliveries by marginalized groups across public and private sectors. It also evaluates the implementation processes that underpin community action and accountability for maternal health in Gujarat, India. The study concurrently draws from qualitative and quantitative data to triangulate data sources to address the aforementioned research aims.

## Methods

### Setting

Gujarat is considered a strong Indian state in terms of administrative capacity and ranks high on economic development. Yet its health indicators are lower than other states that are less economically endowed. Significant gaps in provision and quality of maternal health care services exist in Gujarat [22] (Table 1). Efforts to work with civil society to strengthen community participation and action for health are particularly important given that the state has resources and capacity, but faces challenges in ensuring that services reach those most in need.

**Table 1:** Key maternal and child health indicators for intervention districts, state and national levels

	At least 4 ANC visits (%)	Institutional Births (%)	Births assisted by a doctor/nurse/LHV/ANM/other health personnel (%)	Children age 12–23 months who have received BCG (%)	Children under 5 years who are wasted (weight-for-height) (%)
Anand	78.5	92.6	93.8	99.1	21.7
Panchmahal	51.2	79.6	79.0	63.7	36.3
Dahod	39.2	84.3	82.3	65.3	24.9
Gujarat	70.6	88.7	87.3	87.9	26.4
India	51.2	78.9	81.4	91.9	21.0

Source: National Family Health Survey 4: 2015–2016

From 2012 to 2015, the NGO Society for Health Alternatives (SAHAJ), led the 'Community Action for Maternal Health Project,' implemented in 45 villages with approximately 108,000 people. The project worked in two different contexts in Gujarat: Dahod and Panchmahal districts (remote, tribal populations) as compared to Anand district (more urban and affluent). This NGO-community partnership simultaneously addressed demand and supply side constraints to poor utilization and low quality of services by a) raising awareness of maternal health entitlements, b) supporting community monitoring of services, and c) facilitating dialogue with health providers and other key stakeholders (Table 2).

**Table 2:** Community action for maternal health project aims and activities

Aim	Specific Activities
1. Awareness of entitlements by pregnant women and community	Focus group discussions and participatory methods with women's groups to elicit local understanding and preferences for safe delivery
	Community wide meetings and group-specific meetings.
	Pictorial banner for group discussions (*toran*) and individual poster for woman about entitlements (*mahiti patrika)*
2. Community monitoring of receipt and delivery of services	Home visits with individual women at 8 months pregnancy and postpartum using healthy mother tool (*warli madi* tool*)*
	Monitoring of outreach antenatal services at Village Health and Nutrition Day (VHND) (VHND tool)
	Maternal death tracking to triangulate government tracking
3. Dialogue with stakeholders about gaps identified	Development of report cards of Primary Health Centre (PHC) functioning
	Support of community members during the Maternal Death Review process

### Raising awareness

Community meetings first elicited local perceptions of safe deliveries among women and providers, before developing a common understanding of essential services and entitlements [10, 23]. Subsequent meetings imparted information about these entitlements; raised awareness about nutrition, antenatal checkups, high-risk symptoms, newborn care, immunization; and served as a space to follow up on other health problems, such as tuberculosis [24]. The meetings also built community ownership around village-level health issues and collective decision-making on who would follow up on actions decided upon. In addition, monthly meetings were convened by volunteers for additional problem solving related to the Public Distribution System, water, early childhood care centers (*Anganwadi*), Below Poverty Line cards, access to other entitlements, based on other findings from community monitoring efforts [25].

In an effort to support birth preparedness, a poster, called a *Mahiti Patrika,* was developed to provide information regarding maternal health services and emergency contact numbers of health providers [23]. This poster was displayed on the exterior house walls of all pregnant women visited. Volunteers reported that women and families used the *Mahiti Patrika* to call the numbers listed. NGO project documentation notes that, in addition to dialing 108 (the emergency transport number) for referral transport, pregnant women and family members also used the information on the *Mahiti Patrika* to follow up on immunization for their child and to solicit help from health providers with deliveries, including those that resulted in complications [25].

Another educational resource utilized was a pictorial banner, called a *Toran*, to increase awareness among women of the services provided through outreach antenatal care services. Project staff noted that the cartoons initially used were not understood due to low literacy levels. Instead, photographs were found to be more effective, particularly those that depicted local women and government health providers [25]. Medical officers found it motivating to be depicted in the *Toran* and were reported by NGO members to be more responsive after receiving such positive publicity. NGO respondents also noted that local political parties also used the *Toran* in their campaigns (Interview 54).

### Community monitoring and dialogue with health authorities

The project also developed the *Warli-Madi* (Healthy Mother*)* tool, a short pictorial checklist of maternal health entitlements and services for community members to prospectively track pregnant and postpartum receipt of key services [23]. Trained volunteers visited households twice with the tool, once during a woman's 8th month of pregnancy and again 10–20 days post-delivery [10]. The tool was finalized in early November 2012 after seven months of development and testing, building on women's own perspectives of safe delivery and professional standards of quality care and subsequently further simplified based on a mid-term review assessment [23, 24].

SAHAJ took primary responsibility for collating data from the monitoring tool into the report cards, investing significant time in checking forms and liaising with ANANDI and KSSS about the data. A color-coded system was developed to denote whether levels of service receipt were poor (red), average (yellow) or good (green). NGO staff noted that over time seeing some of the indicators change in color was highly motivating to community members and health providers alike (Interviews 39–41).

In Dahod and Panchmahal districts, the local NGO shared report card results at trainings with volunteers, during women's collectives meetings and ward meetings. Report cards were first shared with local health authorities and medical officers in March 2013. Subsequently, medical officers were interested in receiving primary health center-wise report cards and discussed the results with their staff [23]. Changes documented include the restarting of services (increasing the number of outreach clinics in hard to reach areas, initiating deliveries in a previously defunct facility), repairs that improved the quality of the service environment (fixing leaks and toilets), better relationships between community members and government providers (health trainings by government providers for women's collectives, invitation to NGO partners to attend block level maternal death review meetings), and addressing inappropriate practices (kick-backs between female community level providers and private providers, private hospital not providing services as per the public-private insurance scheme) [25, 26].

### Methods

Qualitative data included project document review (annual reports, presentations, other project material) and 56 interviews undertaken in 2015 with purposively sampled community members (*n* = 22), health providers (*n* = 16), health authorities (*n* = 8), and project personnel from implementing NGOs (*n* = 10).

For the qualitative component, extensive consultations were undertaken with the NGO to collect data from villages across districts that were categorized by the NGO as having strong vs. weak implementation to ensure that diverse project experiences would be captured. Semi-structured interview guides were reviewed with implementing NGOs and piloted before being finalized for use. Interviews were done by four data collectors. A local research assistant, a foreign postgraduate student with family in the region, and two more senior medical doctors with extensive community medicine, civil society and government experience. Interviews were carried out in June–July 2015. Interviews were done in the language the respondent was most comfortable with given that the data collectors were fluent in Gujarati, Hindi and English. While the NGO facilitated transport and introductions to key project personnel and leaders in the villages, written consent was obtained by the interviewers without involvement of the NGO. Interviews were recorded electronically if permission was given and detailed notes were taken irrespective of whether the interview was electronically recorded or not. After listening to the interviews, only those that were the most rich across key stakeholders were transcribed.

The research team developed and pilot tested a code book. The final set of codes were derived from the research aims, the interview guide and from the transcripts following three overarching domains; project processes, outcomes and context. We undertook thematic analysis of interviews applying the codes to the interview notes and transcripts from varied stakeholders at different levels of the health system. Triangulation across project documentation and interviews helped understand project processes more fully. Peer review debriefing between the interviewers and key NGO staff helped improve the quality of the data analysis. Research was planned closely with local NGOs to ensure consideration of the local context and accurate interpretation of findings.

The design of the project was to empower women through the collection, analysis and dissemination of data at village level. Volunteers collected the data on pregnant women prospectively through household visits in their respective villages. Hence the data are not based on any pre-determined sampling design and represent the efforts of volunteers to gather data from as many women as possible. The data collected by the volunteers was then collated and analyzed by the NGO, with a view to sharing results back to the community. For the quantitative data, we analyzed the available data that had been collected by the volunteers through monitoring of pregnant and postpartum women (Dahod and Panchmahal districts, *n* = 1145 pregnant and postpartum women, Anand district *n* = 1250 pregnant and postpartum women). The number of women was determined by the capacity of the village health volunteers to track the women and varied from village to village based on the local volunteers' time availability and motivation. The results presented in the paper use the data collected by the volunteers without any sampling modifications.

In analysing the household level data from the project database, we restricted analysis of indicators to variables in districts with the longest implementation, where definitions were unchanged, and data collection was considered reliable. Frequencies and proportions were used to analyze the joint distribution of predictor variables (like demographic and socioeconomic characteristics) and care seeking variables including awareness of maternal health entitlements, content of antenatal care and care seeking for delivery care across years of implementation.

Multivariable logistic regression was focused on the delivery care seeking variable, whether there were any changes in the pattern of care seeking for delivery care among the most vulnerable groups (Schedule Caste and Schedule Tribes (SC/ST) compared to the less vulnerable groups (General Caste (GC) and Other Backward Castes (OBC)) over the duration of the project. Two indicators for patterns in care seeking for delivery were analyzed: a) proportion of all pregnant women using health facilities for delivery care; b) proportion of women delivering at facilities who use government facilities. Other explanatory variables include mother's education, occupation, ownership of mamta card (maternal and child health card) and type of family. Table 3 describes the different variables used in the regression model. All the independent variables were fit using the multivariable logistic regression model. To model the effect of time (program effect) on different groups of vulnerability, an interaction term of time and dimension of vulnerability (by caste) was added to a logistic regression model. All women were used for the outcome of facility delivery, while the model for type of facility used for delivery was restricted to the women who reported delivering at a facility

$$ probability\kern0.3em of\kern0.34em outcome(y)=b0+b1 vulnerability+b2 time+b3{time}^{\ast } vulnerability+b4\left( Other\mathit{\exp}\mathit{\ln } atory\mathit{\operatorname{var}} iables\right) $$

**Table 3:** Variables used in multivariable regression

Variables used in multivariable logistic regression	Categories
Facility delivery	Delivered at a facility Delivered at home or on the way
Type of facility	Government/public facility Private facility
Mother's education	No schooling Primary Secondary or higher
Mother's occupation	Not employed Employed
Ownership of mamta card (maternal and child health card)	No Mamta card Have Mamta card
Type of families	Joint Nuclear
Time	Year 2013 Year 2014 Year 2015
Vulnerability	Least vulnerable - General Caste and Other Backward Castes Most vulnerable - Schedule Caste and Schedule Tribes

Clustering of responses at the village level was accounted for by the use of robust variance estimators based on a first-order Taylor series linear approximation. The adjusted prevalence of the outcome indicators for Dahod and Panchmahal over the period of the project are presented graphically with 95% confidence intervals. For Anand, the interaction models did not achieve convergence and hence the proportions are presented.

## Results

We first report findings from the prospective household tracking tool detailing project outcomes related to awareness of entitlements and access to facility deliveries. We then report stakeholder perceptions of key implementation features and processes including NGO organizational mission and alignment, extensive capacity building, receptiveness of state actors in terms of acknowledgement and accountability.

### Project outcomes

#### Awareness of entitlements

**Fig. 1:**
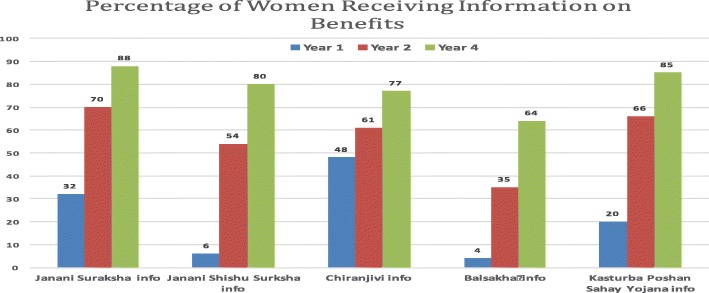
Frequency of receipt of information on entitlements to government programs in community monitoring data, Dahod and Panchmahal districts, Dec 2012-Oct 2015, *N* = 1145

Substantial gains were made in pregnant and postpartum women reporting receipt of information from government providers about key maternal health entitlements to government schemes relevant to their health needs and rights in Dahod and Panchmahal, the more marginalised districts (Fig. 1).

Medical officers and health administrators concurred that project activities had increased awareness of maternal health "they [women] had no idea before" (Interview13). Interviews with women beneficiaries were more nuanced. Some women struggled to articulate rights or accountability as concepts (Interview 14), while others detailed that pregnant women should have good diets, access to entitlements and have safe deliveries at well-equipped PHCs (Interview 5). Although some women could not list project specifics (eg. that a tool was filled) (Interview6), most recalled a household visit from a local volunteer.

As mentioned earlier, not all volunteers interviewed were familiar with the report card. However, some did report seeing it and one volunteer noted how the report card indicated change in services provided by PHCs. While community health providers (Accredited Social Health Activists (ASHAs) and Anganwadi workers (AWWs)) interviewed by the study did not recall the report card (Interview 3; 15), facility-based providers did report reviewing it with NGO staff (Interview 12). Medical officers reported that they found the information useful (Interview 10, 22, 36). Only one district authority reported skepticism about the data asking, "how do they do their survey, [there is] no one from our system." (Interview 49).

#### Access to services

**Fig. 2:**
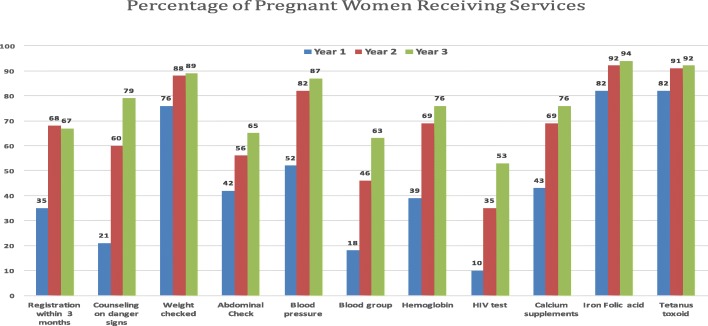
Receipt of ANC services by pregnant women in community monitoring data, Dahod and Panchmahal districts, Dec 2012-Oct 2015, N = 1145

A key outcome of the dialogues facilitated by NGOs based on community monitoring and mobilization was the restarting of services previously suspended. Not only were outreach ANC clinics restarted, but weekly antenatal clinics at facilities were also reported to become more consistent due to the more regular attendance of government providers [25], leading to improved utilization (Fig. 2, Table 4). Medical officers viewed project volunteers as instrumental in improving demand at immunization camps and for facility delivery (Interview 22, 36).

**Table 4:** Multivariable logistic regression of facility delivery ad type of facility for delivery care

	Facility delivery (*n* = 1235)				Government facility delivery (among those who delivered at a facility) (*n* = 542)			
	Odds ratios	*p*	95% CI		Odds ratios	*p*	95% CI	
Year								
2013	1.0	.	1.0	1.0	1.0	.	1.0	1.0
2014	1.7	0.05	1.0	3.0	0.4	0.10	0.2	1.2
2015	1.5	0.25	0.8	2.9	0.3*	0.02*	0.1*	0.8*
Social group								
Least vulnerable	1.0	.	1.0	1.0	1.0	.	1.0	1.0
Most vulnerable	0.7	0.24	0.4	1.2	1.4	0.48	0.6	3.5
Year group interaction								
2014 # Most vulnerable	0.7	0.37	0.4	1.5	3.0*	0.05*	1.0*	8.7*
2015 # Most vulnerable	0.9	0.66	0.4	1.7	9.2***	0.00***	2.9***	29.3***
Possession of mamta card								
No Mamta card	1.0	.	1.0	1.0	1.0	.	1.0	1.0
Have Mamta card	1.8***	0.00***	1.4***	2.4***	2.4*	0.02*	1.1*	5.0*
Woman's occupation								
Not employed	1.0	.	1.0	1.0	1.0	.	1.0	1.0
Employed	0.8	0.10	0.6	1.0	0.7*	0.02*	0.5*	0.9*
Type of family								
Joint	1.0	.	1.0	1.0	1.0	.	1.0	1.0
Nuclear	1.5***	0.00***	1.2***	1.9***	0.8	0.42	0.5	1.3
Woman's education								
No schooling	1.0	.	1.0	1.0	1.0	.	1.0	1.0
Primary	1.9***	0.00***	1.5***	2.5***	0.5**	0.00**	0.4**	0.8**
Secondary or higher	3.3***	0.00***	2.1***	5.1***	0.6*	0.04*	0.4*	1.0*

* 0.05 ** 0.01 *** 0.001

The results of multivariable logistic regression to model factors associated with delivery care at a facility and use of a private facility for delivery care are presented in Table 3. Women with a Mamta card, belonging to a nuclear family, and educated to primary and/or secondary school of higher, were more likely to report delivering at a facility. With regards to the public private split, educated and employed women were less likely to deliver at government or public facilities compared to uneducated and unemployed women. Women with a Mamta card more likely to report delivering at government or public facilities than those without. The regression models were used to predict adjusted marginal proportions for the outcome variables.

**Fig. 3:**
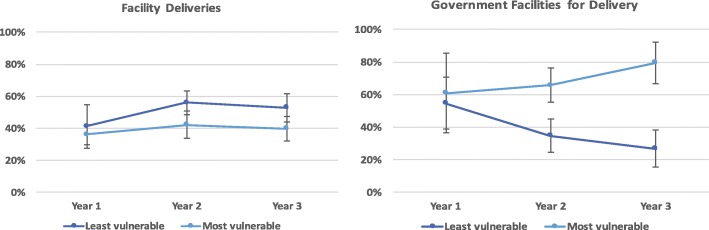
Extent and location of facility deliveries for the least vs. most vulnerable (SC/ST) from year 1 to year 3 adjusted for socio-demographic variables in community monitoring data, Dahod and Panchmahal districts, Dec 2012-Oct 2015, N = 1145

**Fig. 4:**
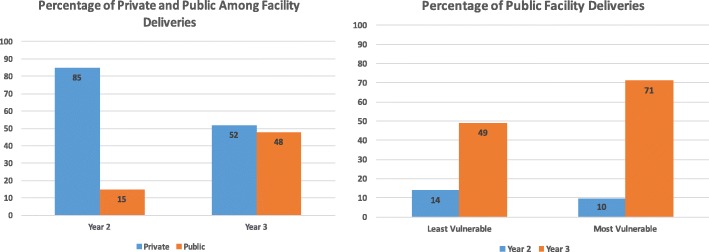
Location of facility deliveries for the least vs. most vulnerable (SC/ST) from year 2 to year 3 in community monitoring data, Anand districts, Jan 2014-Oct 2015, *N* = 1250

With regards to facility deliveries, the proportion of women delivering at facilities increased overtime (33% to 53%) in Dahod and Panchmahal, the more marginalized districts. While the overall increase in facility deliveries did not have equity effects, the change in type of facility did. The proportion of women delivering in government vs. private facilities increased for the most vulnerable group and decreased for the least vulnerable group (Fig. 3). In Anand district, access to ANC and facility deliveries was already very high at the beginning of the project. Within a year of the project, the location of facility deliveries changed towards favoring government centers, particularly for vulnerable populations (Fig. 4).

### Project processes

#### Organizational mission and alignment

**Fig. 5:**
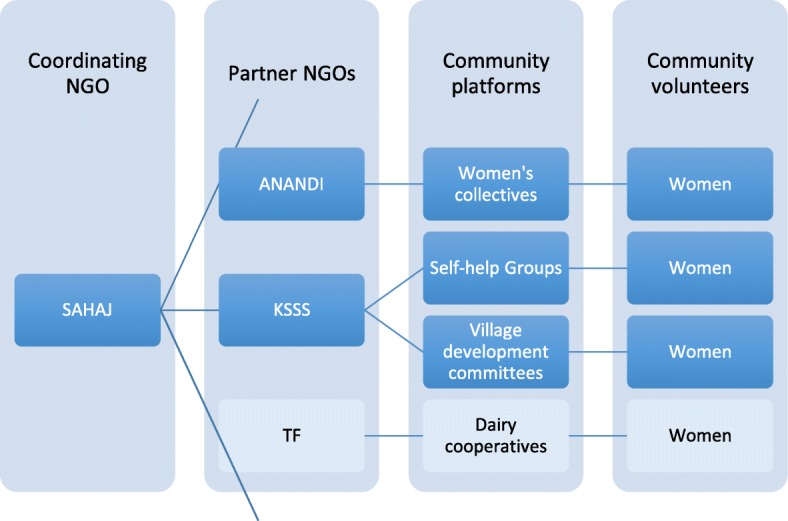
Community action for maternal health implementing organizations and structures

The community action for maternal health project led by SAHAJ facilitated implementation of project activities through three community-based organizations: Area Networking and Development Intitiatives (ANANDI), Tribhuvandas Foundation (TF) and Kairo Social Service Society (KSSS) and their corresponding community platforms (women's collectives, self-help groups, village development committees and dairy cooperatives) (Fig. 5). The varying mission and history of these four partners affected their involvement and the outcomes. NGO staff from all partners indicated that they were motivated to work on the project, because they recognized limits in their prior efforts to address the needs of marginalized pregnant women (Interviews 40,41). Nonetheless, one NGO saw itself primarily as a service delivery organization. Their discomfort with confronting government with which they had previously built a good relationship was part of the reason why they withdrew from project (Interview 54). In contrast, the mandate of the other NGOs was to empower collectives of poor and marginalized women and build capacity for rights-based approaches. In particular, volunteers drawn from ANANDI with their history of supporting women's collectives, reported more confidence and required less capacity building than volunteers from the other NGOs. In addition, while all partners experienced a high turnover in volunteers, NGO staff felt that the ANANDI women's collectives were more able to sustain continuity of project activities (Interview 54).

#### Extensive capacity building

Significant time was spent training volunteers on how to use the tool. Volunteers collecting data with the tool reported initially finding the work challenging due to self-doubt, asking, "how will I give information, how will I fill the tool?" (Interview21). In addition, volunteers reported that they had to overcome skepticism about the purpose of the tool and the information they were meant to convey, as some families perceived volunteers to be paid for collecting the information, that they would receive benefits after helping volunteers complete the tool or that volunteers would steal the benefits linked to the tool (Interview 30).

An underlying assumption behind developing the tool was that it should serve as more than just a monitoring mechanism. The home visits and dialogues facilitated while filling out the tool were meant to raise awareness among women and families. In practice, volunteers and project staff were very concerned with the completion of the tool and the quality of the data within it (Interview 7, 54). Interviews with pregnant women did not reveal familiarity with the monitoring tool (Interview 5) and project volunteers mainly reported sending the information to NGO staff for processing (Interview 30). Knowledge about the report card and use of data for health system engagement was variable among women and project volunteers (Interview14). Some volunteers had extensive knowledge (Interview 53) whereas others confused the report card with a malnutrition chart (Interview 7). However, women, project volunteers and NGO staff all concurred that broader awareness of maternal health services and entitlements was raised through this process, even if women did not recognize the specifics of the monitoring tool itself and project volunteers were not involved in compiling or generating the report cards (Interview 21, 33, 40).

Reflecting on the tool, NGO staff reported that developing the tool in a participatory manner was essential for local ownership*.* Nonetheless, none of the NGO staff had anticipated the extent of time and capacity building that the tool absorbed (Interview 41, 54). This was another reason why TF withdrew from the project (Interview 54). In terms of next steps, NGO staff felt that further work was needed for the tool to be owned by women and communities more directly. Currently, NGOs process the information in the tool and lead the dialogue with health providers and authorities. NGO staff perceived that a shorter tool that allows women to self-identify services not being provided and to facilitate change using the appropriate contact information given, may enable further empowerment and local ownership (Interview 39,41).

#### Acknowledgement and accountability

The NGOs involved were valued for instrumental reasons by health authorities at both the district and block level, as they were working to "understand what public expectations are and what government services are available" (Interview 45). Anand district-level authorities mentioned periodically receiving monitoring results from the NGOs and noted that having extra hands to work on the ground helped (Interview 45). At the same time, the research team when interviewing community level, female government providers, felt notable tension. This was attributed in the debriefing to the NGOs monitoring their performance and advocating for greater responsiveness from these community level providers. While valuing the NGOs for improving health awareness and utilization, district and block health authorities did not see the need for accountability to communities (Interview 46). Their prime concern was NGO aid and support. When discussing ideas for future plans, they mainly suggested a focus on awareness (Interview 45) or geographic expansion and coverage of more marginalized populations (Interview12). In contrast, state health authorities more directly discussed the need for community accountability, indicating that community monitoring experiences from other states had shown a role in improving health care services, particularly if led by independent NGOs (Interview 51,52).

## Discussion

NGO facilitated community-based action addressed demand and supply side constraints to care seeking for maternity services through three key strategies: raising awareness, community monitoring, and dialogue with government health providers and authorities based on report cards. Supportive implementation factors included alignment with organizational mission, participatory development of project tools, repeated investment in capacity building, NGO facilitation of community monitoring and dialogue with authorities, government interest in improving utilization and NGO legitimacy due to their contributions. Initial challenges included confidence and turnover of volunteers, complexity of the monitoring tool and scepticism from both communities and providers. While not all women were monitored by volunteers and the extent of community involvement in the monitoring and dialogue with government providers and authorities was not as broad as originally planned, ongoing facilitation of community action by NGOs did accomplish important gains in terms of health awareness and utilization of services, particularly for the most marginalized. Even among the wealthier and urban district, there was a switch from private to public deliveries for the most vulnerable.

While evaluations have demonstrated the impact of similar initiatives on care seeking and mortality [7], few have evaluated equity impacts or changes in care seeking across public and private sectors. In another state in India, providing information on entitlements without additional community action improved care seeking but failed to change health inequities in such care seeking [8]. Our results not only show improvements in Dahod and Panchmahal districts where care seeking was previously very low, but also significant changes in the public private mix in care seeking in Anand district, where care seeking was previously high.

Before further expansion, greater investment is required to strengthen the support systems and capacity building required to ensure that more women in targeted communities are reached and that sufficient capacity among community groups and NGO staff exists. Changing the internalized social norms that marginalize disadvantaged women takes time but can yield deep dividends [12, 13]. Alignment with NGOs that have extensive experience in selected communities [27, 28] and that have an organizational ethos aligned with community action and accountability initiatives is critical for fostering relationships of trust, reach, credibility and constructive dialogue needed for the project [12].

The NGOs involved felt that the monitoring tools could be further simplified to enable volunteers to analyze the data collected and engage more directly with the dialogue processes. Significant time was spent on training, reviewing and revising the monitoring tools. While the use of report cards that visibly tracked change brought legitimacy to NGO and community demands, rather than attribute this purely to the objectification of data and tools, the social processes underpinning such change needs further examination [29]. The project assessment also revealed nuances in levels of community engagement with the project. While the project focused on community action, this was primarily driven by the local NGO and the community volunteers. How extensively does the broader community need to be engaged to deliver improvements in care seeking [30]? How does this vary by type of community intervention (information dissemination alone vs participatory action initiatives) and nature of platform (women's collectives, health committees, self-help groups)? Should initiatives to improve government service delivery and potentially monitor the private sector rely women from marginalized communities volunteering their time? While there is a consciousness that it is unfair for health systems to rely on unpaid female community volunteers [31, 32], similar introspection has yet to cross over into the social accountability field.

Constant dialogue is needed with government providers and authorities [33, 34]. Tensions particularly with frontline providers whose performance is being monitored and called into question needs to be negotiated so that, leaving aside egregious errors, the structural constraints that inhibit service delivery and its quality is addressed [35, 36]. Community and NGO initiatives in monitoring access to services with the express intent of addressing marginalized women's needs and entitlements proved to be an important starting point for dialogue with providers on how to improve service delivery. Acknowledgement and cooperation across health system levels from government health providers to higher level authorities is critical for these dialogues to translate into effective action [34, 37, 38]. While stakeholders may come from different starting points, building a common understanding and momentum for change is critical [28, 39].

The study relied on prospective project monitoring data that did not completely cover all pregnant women in the project area, although it did cover women living in more marginalized areas. Data contrasting comparison and intervention areas from government information systems could be used if project villages aligned with government administrative areas more closely. While qualitative interviews were undertaken with project stakeholders across health system levels, those at community level were challenging to undertake. More participatory approaches to gauging community perceptions and priorities require more time, but may have been more revealing.

## Conclusion

Over three years, NGOs supported community action for maternal health, investing significant capacity building and facilitation to foster improved mutual understanding, trust and collaboration across disparate actors across various levels of the health system in rural and urban Gujarat, India. Despite the challenges faced, the project contributed to increased awareness of maternal health entitlements, increased utilisation of antenatal and of government facilities for institutional deliveries particularly among the marginalized, and improved accountability of services. Over time, with flexible resources and iterative adaptations and learning, capacity can be developed in community-based organizations and platforms to monitor health services and engage with front level providers, with important results for utilization and equity.

## Abbreviations

ANANDI: Area Networking and Development Intitiatives
ASHAs: Accredited Social Health Activists
AWWs: Angan wadi workers
GC: General Caste
KSSS: Kairo Social Service Society
NGOs: Non-Government Organizations
OBC: Other Backward Castes
SAHAJ: Society for Health Alternatives
SC/ST: Schedule Caste and Schedule Tribes
TF: Tribhuvandas Foundation
VHND: Village Health and Nutrition Day
